# Novel methodologies and technologies to assess mid-palatal suture maturation: a systematic review

**DOI:** 10.1186/s13005-017-0144-2

**Published:** 2017-06-14

**Authors:** Darren Isfeld, Manuel Lagravere, Vladimir Leon-Salazar, Carlos Flores-Mir

**Affiliations:** 1 Orthodontic Graduate Program, School of Dentistry University of Alberta, Edmonton, AB Canada; 2 School of Dentistry, University of Alberta, Edmonton, 11405 - 87th avenue, Edmonton, AB T6G 1C9 Canada; 3 Division of Pediatric Dentistry, School of Dentistry, University of Minnesota, Minneapolis, MN USA; 4 Orthodontic Graduate Program, School of Dentistry, University of Alberta, Edmonton, AB Canada; 5 School of Dentistry, University of Alberta, Edmonton, AB Canada

**Keywords:** Cone-beam computed tomography, Palatal suture, Maxillary expansion

## Abstract

**Introduction:**

A reliable method to assess midpalatal suture maturation to drive clinical decision-making, towards non-surgical or surgical expansion, in adolescent and young adult patients is needed. The objectives were to systematically review and evaluate what is known regarding contemporary methodologies capable of assessing midpalatal suture maturation in humans.

**Methods:**

A computerized database search was conducted using Medline, PubMed, Embase and Scopus to search the literature up until October 5, 2016. A supplemental hand search was completed of references from retrieved articles that met the final inclusion criteria.

**Results:**

Twenty-nine abstracts met the initial inclusion criteria. Following assessment of full articles, only five met the final inclusion criteria. The number of subjects involved and quality of studies varied, ranging from an in-vitro study using autopsy material to prospective studies with in vivo human patients. Three types of evaluations were identified: quantitative, semi-quantitative and qualitative evaluations. Four of the five studies utilized computed tomography (CT), while the remaining study utilized non-invasive ultrasonography (US). No methodology was validated against a histological-based reference standard.

**Conclusions:**

Weak limited evidence exists to support the newest technologies and proposed methodologies to assess midpalatal suture maturation. Due to the lack of reference standard validation, it is advised that clinicians still use a multitude of diagnostic criteria to subjectively assess palatal suture maturation and drive clinical decision-making.

**Electronic supplementary material:**

The online version of this article (doi:10.1186/s13005-017-0144-2) contains supplementary material, which is available to authorized users.

## Background

Rapid maxillary expansion (RME) is indicated for a number of clinical situations namely when a posterior crossbite exists (unilateral or bilateral) or limited buccal overjet in patients with constricted maxillary base [1]. Maxillary transverse deficiency may be skeletal, dental or both skeletal and dental in origin [1–3]. Expansion in the transverse dimension has not only been used to improve interdigitation of the occlusion and improved function but also to increase arch perimeter to resolve maxillary crowding [2]. Recently contemporary orthodontics has focused on smile esthetics with emphasis on transverse arch dimensions and minimizing buccal corridor visibility [1, 4]. Those patients with dentofacial deformity or cleft lip and palate with constricted maxillary segments are candidates for RME or possible surgical expansion [2] dependent upon the time of treatment intervention. Additionally, there has been increased interest in the use of RME to increase nasal airway volume and/or function [1, 2].

Treatment options available to clinicians for maxillary expansion include tooth-borne expanders with or without an acrylic support [2, 5], bone-borne maxillary expansion devices supported by temporary (skeletal) anchorage devices [5], as well as surgically assisted rapid palatal expansion [1, 3]. The treatment of choice is dependent on numerous clinical indications including; the extent of correction required, whether skeletal or dentoalveolar correction is indicated, and perceived efficacy of expansion based on timing of treatment [6].

The amount of skeletal or dentoalveolar effect of the RME is directly correlated with the stage of skeletal maturation of the palatal suture. Treatment timing of transverse deficiencies is recommended relatively early up to peak skeletal growth velocity [6]; however, there is significant variation in the timing of skeletal maturation amongst individuals [2, 6] as the palatal suture fusion is poorly correlated with patient age and sex [3]. Failure to properly identify key clinical signs and provide individual assessment to identify a patient’s ideal expansion treatment option can lead to iatrogenic side effects and co-morbidities [3, 6]. Common side effects of poorly timed and failed conventional RME therapy include acute pain [2], gingival recession, dehiscence formation, palatal mucosa necrosis, buccal dentoalveolar tipping and poor long term expansion stability [3, 6]. Conversely prematurely committing a patient to surgically assisted expansion ascribes a patient to a potential significant burden of treatment including increased cost, pain and healing time.

Numerous methodologies have been proposed to discern the architecture and degree of palatal suture fusion including animal and human histologic studies, evaluation of occlusal radiographs, and CT of both autopsy material and animal specimens [3]. Such methodologies presented inherent difficulties in assessing the degree of palatal suture fusion. As defined previously, histological evaluation is the reference standard to evaluate midpalatal suture maturation, unfortunately implementation on active orthodontic patients would require an invasive biopsy, precluding its use [7, 8]. Conversely, serial occlusal radiographic assessment is limited in diagnostic quality due to superimposition of nearby anatomical structures [3]. Cone-beam CT (CBCT) allows for 3D rendering of the maxillofacial complex without superimposition of nearby anatomy and delivers a lower absorbed dose of radiation to the patient than medical CT [3]. To date, however, there has been no validated non-ionizing method to assess palatal suture maturation.

The objectives of this systematic review are to thoroughly describe and evaluate the contemporary technologies and methodologies capable of assessing midpalatal suture maturation.

## Methods

The Preferred Reporting Items for Systematic Reviews and Meta-Analysis (PRISMA) statement checklist was followed; however, several points did not apply to this systematic review. This is a review of both in vitro and in vivo studies rather than solely in vivo studies, convoluting the direct comparison of results amongst these types of studies and their possible clinical inferences. No protocol registration was done.

### Eligibility criteria

Both in vitro and in vivo studies will be included to identify all diagnostic modalities of palatal suture maturation. The intervention(s) will be any diagnostic method that is designed to evaluate the degree of ossification and/or interdigitation of the midpalatal suture (the outcome). Comparison will be to other diagnostic interventions designed to evaluate the same outcome variable.

The “participants” will be any human subjects or human specimens being investigated for the degree of midpalatal suture maturation. No animal studies were considered as their applicability in humans would be questionable.

### Information sources

A computerized database search was conducted using Medline, PubMed, Embase and Scopus to search the literature ranging from 1980 up until October 5, 2016. A supplemental hand search was completed of references from retrieved articles that met the final inclusion criteria.

### Search

Terms and their respective truncations used in the literature search (Appendix 1) were specific to each database. Searches were conducted with the help of a senior librarian who specializes in the health sciences. The selection process was carried out together by two researchers (DAI and HE). All references were managed by reference manager software EndNote to eliminate duplicates.

### Study selection

The inclusion criterion “*Diagnostic methods to evaluate cranial suture ossification/maturation*” was utilized to initially identify possible articles from the published abstract results of the database search. If an abstract was not available, the full text was reviewed for appropriateness of inclusion. Any disagreement on the inclusion of a study was resolved by discussion amongst the reviewers.

Once these abstracts were selected, full articles were retrieved and inclusion in the systematic review was dependent of fulfilling a final inclusion criterion. The final selection criterion was as follows: “*In vitro and in vivo human subject studies that describe a novel diagnostic method or technology to assess midpalatal suture maturation/ossification over time”.* Once more, any disagreement on the inclusion of a study following this final criterion was resolved by discussion amongst the reviewers. The references cited in the finally selected articles were also screened for any applicable references missed in the electronic database search.

Studies describing diagnostic methodologies applied to theoretical models without practical application were excluded. One article was excluded since no German translation was obtained. No other language restrictions were applied.

### Data collection process

Data extraction was performed and collected by a researcher (DAI).

### Data items

The variables collected included a description of the type of study, type and number of subjects, study objectives, inclusion criteria, imaging modality used, region(s) investigated, and methodology to evaluate degree of ossification/maturation of midpalatal suture (Tables 1 and 2).

**Table 1:** Summary of articles that met final inclusion criteria

Author(s)	Franchi et al. [11]	Sumer et al. [9]	Korbmacher et al. [10]	Angelieri et al. [3]	Kwak et al. [12]
Type of Study	Prospective study	Prospective study	In-vitro study	Cross-sectional	Cross-sectional
Human Subjects or Material	Human subjects	Human subjects	Human autopsy material	Human Subjects	Human subject
Study Objective(s)	Assess the midpalatal suture density via lowdose computed tomography (CT) prior to RME (T0), at the end of active RME (T1), and following a 6 month retention period (T2).	Evaluate the efficacy of ultrasonography (US) to generate a qualitative assessment of ossification post-SARME.	Quantification of sutural morphology via micro-CT and its association with age.	To validate and present a novel classification system for the individual assessment of midpalatal suture morphology using CBCT.	Evaluate the correlation of fractal patterning to ossification of the palatal suture via CBCT evaluation and determine whether fractal analysis of the midpalatal suture can be used to assess the maturation of the suture.
# of Subjects and Inclusion Criteria (if applicable)	17 patients, 7 male, 10 female, mean age of 11.2 years old, range of 8–14 years old. Inclusion criteria: patients with constricted maxillary arches with or without unilateral or bilateral posterior crossbite, and within cervical vertebral maturation (CS1-CS3)	3 patients, bilateral transverse maxillary deficiencies requiring SARME. Age, sex and developmental characteristics of subjects not given.	28 human-palate specimens, (11 female, 17 male) aged 14–71. The palatal specimens were categorized by the donor’s age into age groups (< 25 years, 25 years to <30 years, ≥ 30 years).	140 subjects (86 female, 56 male), age range from 5.6 to 58.3 years old, Inclusion criteria: patients who are undergoing initial records for orthodontic treatment and who have received no previous orthodontic treatment.	131 subjects, (69 men and 62 women), mean age mean age of 24.1 ± 5.9 years (male subjects 23.1 ± 5.8 years, female subjects 25.2 ± 5.9 years) Age range of18.1–53.4 years old. No specific inclusion criteria noted
Study’s Expansion Modality, Expansion protocol, Average amount of Expansion (mm)	Modality: butterfly palatal expander Protocol: standard protocol – activated twice per day (0.25 mm per turn) for 14 days. Retention period of 6 months than appliance removed. Amount of expansion: 7 mm in all subjects	Modality: SARME (tooth borne Hyrax). Protocol: 0.8–0.9 mm expansion/day in two daily activation steps until desired expansion achieved, ~14 days. Retention period of 6 months, then hyrax removed. Amount of expansion: not specified but based on clinical needs of patient.	Not applicable, no expansion performed.	Not applicable, no expansion performed.	Not applicable, no expansion performed
Imaging Modality	Multi-slice low-dose Computed tomography (brand information not given). Standardized axial CT images parallel to the palatal plane and passing through the furcation of maxillary right first molar, scans acquired and magnified (3×) with Light-Speed 16 software (General Electric Medical System, Milwaukee, WI).	Color-coded Ultrasonography duplex scanner (Aplio 80, Toshiba Tokyo, Japan) with 7.5-MHz linear-array transducer	Scanco Micro-CT 40 (Scanco Medical, Bassersdorf, Switzerland) 70 kV, 114 μA. Isotropic voxel size 37 μm. Maximum scanning time of 200 min/specimen. Data analyzed using V4.4A software (Scanco Medical, Bassersdorf, Switzerland). 3D reconstruction via AMIRA 3.00 software m(TGS, Mercury Computer Systems, San Diego, CA). Bone volume and quantification via Image Tool 3.00 software (UTHSCSA, San Antonio, TX),	iCAT cone-beam 3-dimensional imaging system (Imaging Sciences International, Hatfield, PA). 11 cm Minimum FOV. Scantime from 8.9 to 20 s resolution of 0.25 to 0.30 mm. Image analysis using Invivo5 (Anatomage, San Jose, CA). A standardized protocol to isolate axial maxillary cross-sections of the palate was presented.	Cone Beam Computed Tomography (CBCT) (Zenith 3D; Vatech Co., Gveonggi-do, Korea) Field of view 20 × 19 cm; voltage 90 kVp; current 4.0 mA; scan time 24 s). Images were assessed using CT software (Ez3D 2009; Vatech Co.),
Region(s) Investigated	Midpalatal suture and maxilla. 4 regions of interest (ROIs); 1. Anterior sutural ROI (AS ROI): located on the suture 5 mm anterior to nasopalatine 2. Posterior sutural ROI (PS ROI): on suture 5 mm posterior to the nasopalatine duct 3. Anterior bony ROI (AB ROI): control ROI on maxillary bone 3 mm to the right of laterally AS ROI 4. Posterior bony ROI (PB ROI): control ROI on maxillary bone 3 mm right of PS ROI	Midpalatal suture	Midpalatal suture	axial central cross-sectional slices generated and used for assessment of the midpalatal suture	axial central cross-sectional slices generated and used for assessment of the midpalatal suture. A long and narrow region of interest within the final axial slice highlighting only the suture was considered for fractal analysis, such that the incisive canal was not incorporated, but rather the ROI extended from posterior to the incisive canal to just anterior to the posterior nasal spine.
Method of Measurements (units)	1 trained and blinded operator (R.L.) calculated bone density values in Hounsfield units (HU). RL performed measurements and repeated all measurements 1 month later. Bone density changes from T0 through T2 at AS ROI and PS ROI contrasted with the Friedman repeated measures ANOVA on ranks and Tukey post-hoc test (SigmaStat 3.5, Systat Software, Point Richmond, CA).	Ultrasonography findings rated via a semi-quantitative bone fill score (0–3). 0 = complete through-transmission of the ultrasound waves, clear gap margins, and no echogenic material; 1 = partial through-transmission of the ultrasound waves, identifiable gap margins, and less than 50% echogenic material; 2 = partial through-transmission of the ultrasound waves, partially obscured gap margins, and greater than 50% echogenic material; 3 = no through transmission of the ultrasound waves, invisible gap margins, and 100% echogenic material. Scores were not supported by histology or CT.	Quantification of 3D Suture Morphology in frontal plane measured: calculated Obliteration index [%], and mean obliteration index [%]. Quantification of 3D Suture Morphology in Axial plane: measured suture length [μm]: linear sutural distance [μm]: interdigitation index;	Definition of the proposed palatal suture maturational stages (A-E) determined by two operators. The definition of each palatal suture maturational stage derived from the histological appearance of suture described in previous histologic studies.	1 principal investigator trained in the Angelieri et al. [3] method categorized the midpalatal sutures of the patients, and the findings were considered the “ground truth” not “gold standard”. Images were reclassified 2 days later two other operators classified 30 images to determine interexaminer reliability. For Fractal analysis, image software (Photoshop CS6 Extended; Adobe Systems, San Jose, CA) was utilized to perform Gaussian blurring and subtract this blurred image from the original, followed by skeletonizing of the binary image, and utilizing the box counting method to determine the fractal dimension. Weighted kappa coefficient was calculated to determine inter- and intra-examiner reliability using MedCalc version 12.3.0 (MedCalc Software, Oostende, Belgium). Fractal dimension at each maturation stage determined by Scheffe’s ANOVA test. Spearman’s correlation coefficient was calculated to determine the correlation between the fractal analysis and maturation stage. Utilized IBM SPSS Statistics version 21.0 software (IBM Co., Armonk, NY) *P* < 0.05 was considered statistically significant.
Measurement time points	Three time points; Before RME (T0), at the end of RME (T1), and after the 6 month retention period	5 time points; after RME, at 2and 4 months during the expansion period, 6 months later where appliance removed and 2 months post appliance removal. Note opening of midpalatal suture confirmed by plain radiograph after active expansion.	One time point evaluated	Single time point evaluated prior to RME. Palatal maturational stage reclassified 2 days later for each patient.	Single time point Palatal maturational stage reclassified 2 days later for each patient.) .

**Table 2:** Results and conclusions of articles meeting final inclusion criteria

Author(s)	Franchi et al. [11]	Sumer et al. [9]	Korbmacher et al. [10]	Angelieri et al. [3]	Kwak et al. [12]
Result(s)	Bone density in the AS ROI and the PS ROI at T0 (563.3 6183.2 HU and 741.7 6167.1 HU, respectively) were significantly smaller than values in the AB ROI and the PB ROI at T0 (1057.5 6129.4 HU and 1102.8 6160.9 HU, respectively). At T0 there was a significant difference in bone density at AS and PS ROIs, but no difference at T1 and T2. AS and PS ROIs showed significant decreases in density from T0 to T1, significant increases from T1 to T2, and no statistically significant differences from T0 to T2.	No statistics reported. Immediately post expansion all 3 patients had a bone fill score = 0. At 2 and 4 months of expansion there was low echogenicity in the suture (US bone fill score = 1) for 2 of 3 subjects. The remaining patient had a bone fill score = 2 at 2 and 4 months respectively. At 6 months post expansion and 2 months after expander removal, 2 of the 3 patients showed a qualitative increase in echogenic material in the suture was seen but less than 100% therefore had a bone fill score = 2, and the remaining patient demonstrated 100% echogenic material, bone fill score = 3. All trends in scores over time were qualitatively confirmed with plain radiographic images.	Frontal plane: No age dependent significance was found for the mean obliteration index (*P* = 0.244). The mean obliteration index was low, varying in all groups (minimum 0%; maximum 7.3%). Middle-aged group’s mean obliteration index tended to be higher than that of either the younger or older age groups but no significant difference was calculated. The highest mean obliteration index (of 7.3%) was found in a 44-year-old male. The oldest individual with a mean obliteration index of 0% was a 71-year-old female. At least one frontal slice per palate – even in the oldest age group – exhibited a suture completely open cranio-caudally. Axial plane: No significant differences detected in all age groups regarding means and standard deviations for suture length, linear sutural distance, and interdigitation index. Interdigitation index computed revealed no significant age-dependent differences (*P* = 0.633). High standard deviation values for suture length, linear sutural distance and interdigitation index were seen in the <25 yo group and >30 yo group, while the 25–30 yo group had far less variation Mean error of measurement amounted to 0.12% for the obliteration index, 2.4% for the suture length, and 0.41% for the linear sutural distance.	The intraexaminer and interexaminer reproducibility values demonstrated agreement, with weighted kappa coefficients from 0.75 (95% [CI], 0.57–0.93) to 0.79 (95%CI, 0.60–0.97), and the reproducibility of examiners with the ground truth demonstrated agreement with weighted kappa coefficients from 0.82 (95% CI, 0.64–0.99) to 0.93 (95% CI, 0.86–1.00). From the 140 subject sample, stage A was observed in children from 5 to approximately 11 years of age, a 13 year old boy was the sole exception. Should be noted there was no fusion of the palatal suture in subjects aged 5 to almost 11 years old. Stage B was observed primarily up to 13 years of age but also 6 of 32 subjects (23% of boys, 15.7% of girls) aged 14 to 18 years old. Stage C primarily depicted from 11 to 18 years of age, with exception being two 10-year-old girls (8.3% of girls) and 4 of 32 adults (15.7% of girls, 7.7% of boys). Stage D was observed in 1 of 24 girls aged 11- <14 years old, and 3 of 19 girls aged 14–18 years old, as well as in 3 of 13 males aged 14–18 years old and >18 years old respectively. Stage E was observed in 5 of 24 females aged 11- < 14 years old and 8 of 19 females aged 14–18 years old and 8 of 19 females aged >18 years old. Stage E was observed in far less males, approximately 9 of 13 males aged >18 years old only.	The intra- and inter-examiner reliability analysis demonstrated agreement for fractal dimension, with a weighted kappa coefficient of 0.84 (95% [CI] 0.74–0.93) and 0.67 (95% CI 0.38–0.95) to 0.72 (95% CI 0.48–0.97) respectively. No subjects had a CVM of 1-IV nor maturational stage A present. 13 of 21 subjects with CVM V were found to have maturational stage B or C (61.9%; males 77.8%, females 50.0%). 42 of 110 subjects with CVM VI were found to have maturational stage B or C (38.2%; males 41.6%, females 34.0%). Post-hoc analysis demonstrated that maturational stages B, C, D and E were related to differences in mean fractal dimension (*P* < 0.05). A negative correlation existed between fractal dimension and maturation stage (−0.623, *P* < 0.001). Male and Female correlation coefficients determined to be −0.649 (*P* < 0.001) and −0.569 (*P* < 0.001) respectively. A receiver operating characteristic (ROC) curve determined the boundary between maturation stages A–C and D or E. Fusion of palatal suture was determinable as a fractal dimension. Fractal dimension is a statistically significant indicator capable of predicting dichotomous maturation stages ((A, B, & C) vs. (D or E) (area under ROC curve [AUC] = 0.794, *P* < 0.001). At optimal fractal dimension cut-off value of 1.0235, statistical analysis to evaluate the predictive ability of fractal analysis to determine maturation stage ((A, B, & C) vs. (D or E)), noted the following values; specificity 86.6%, Sensitivity 64.9%, false positive rate 35.1%, false negative rate 13.4%, positive predictability 80.3%, and negative predictability 74.6%.
Conclusion(s)	Prepubertal subjects demonstrated a lower bone density at the mid palatal suture as compared to the lateral control ROIs on ossified maxillary bone. The post-expansion low bone density at the sutural ROIs supported findings that prepubertal RME effectively opens the suture. Six months of retention following RME allows reorganization and ossification of the midpalatal suture with sutural bone density values similar to pre-RME values.	Ultrasound bone fill scores increased directly with the duration of time post active expansion (authors referred to this as part of the expansion period) Non-invasive US can yield accurate information regarding bone formation at the midpalatal suture in patients undergoing SARME.	Authors note Micro CT analysis disproves the hypothesis of progressive closure of the suture directly related to patient age. Skeletal age and/or calculation of an obliteration index is not useful in terms of diagnostic criteria to drive clinical decision making regarding the perceived efficacy of non-surgical RME. Micro-CT Quantification of the midpalatal suture yields very low obliteration and age- independent interdigitation in the coronal plane. All calculated parameters demonstrated substantial inter-individual and intra-sutural variation.	Utilizing CBCT to assess the midpalatal suture avoids any overlapping of soft and hard tissues. Authors note that their proposed methodology may be useful in reliably driving clinical decision making as it relates to pursing a non-surgical (RME) or surgical expansion intervention (SARME).	Adult patients possess a greater proportion of non-fused palatal sutures than what is assumed. Therefore age of the patient should not drive SARME initiation. Authors report a significant correlation between fractal dimension and degree of maturation of the midpalatal suture Determination of the fractal dimension cut-off value could be used as a reference to pursue RME vs. SARME Fractal analysis can be utilized to evaluate the degree of maturation at the palatal suture.

### Summary measures

The outcome measures included quantitative and/or qualitative results attained with applicable units to describe bone density, ossification or maturation of the palatal suture.

### Synthesis of results

As the data was not considered homogeneous enough a meta-analysis was not conducted.

## Results

### Study selection

**Fig. 1:**
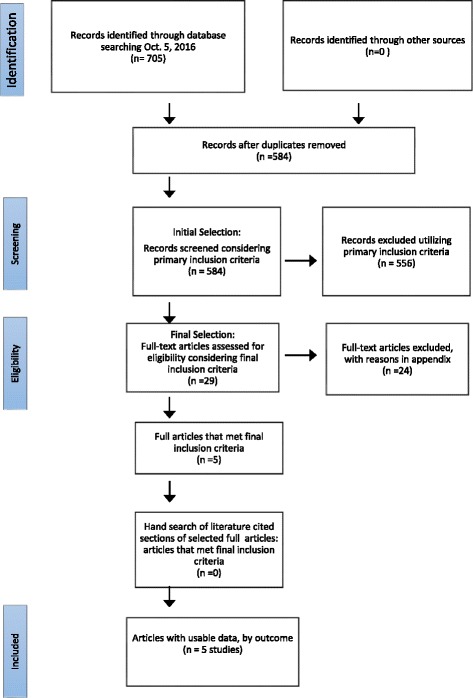
Flow diagram of the literature search

Twenty-nine abstracts met the initial inclusion criteria. Following retrieving of the full articles, only five met the final inclusion criteria. Reasons for exclusion due to final inclusion criteria are stated in Additional file 1. A hand-search of the reference lists from the articles that met the final inclusion criteria identified no new articles. Therefore, a total of five articles were finally considered (Fig. 1).

### Study characteristics

The methodology of each selected article was summarized in Table 1 and results in Table 2. Study parameters, including the type of study, imaging modality used, methodology to determine the ossification/maturation of the palatal suture and the number of subjects amongst other variables were vastly different amongst the studies meeting the final inclusion criteria.

The studies varied significantly in the number of subjects evaluated and quality of evidence. The studies ranged from having three human subjects in a prospective study [9] to 140 human subjects in a cross-sectional study [3]. The types of studies ranged across the hierarchy of evidence from an in-vitro study [10] to prospective in vivo studies [9, 11].

The only study characteristic common to all studies was the region of interest (ROI) investigated, generally speaking, the maxilla. Four of the five studies [3, 9, 10, 12] had a single common ROI which was the palatal suture. One study [11] evaluated four ROIs in the palatal suture and surrounding hard tissue.

All studies but one utilized CT in some form. The types of CT scanners utilized in the four studies included multi-slice low-dose computed tomography (brand information not given) [11], dental CBCT [3] and the extremely high resolution Micro-CT [10]. One study [9] utilized a less invasive modality of US, specifically using color-coded US duplex scanner (Aplio 80, Toshiba, Tokyo, Japan).

To measure the degree of maturation/ossification at the palatal suture, one of three types of evaluations were utilized amongst the five studies: quantitative, semi-quantitative and qualitative.

Franchi et al [11] performed a quantitative evaluation of the palate using one blinded operator to calculate the radiodensity (Hounsfield units [HU]) of the ossification at the palatal suture from T0 (pre-expansion) and T2 (at 6 months retention).

Korbmacher et al. [10] also performed a quantitative evaluation of sutural maturation by measuring the maturation of the palate cadaver specimens at one time point. In the coronal plane, an obliteration index (%) and mean obliteration index (%) was calculated by comparison of the total length of the suture to the length that has ossified (evaluated every 370 μm). The degree of interdigitation of the palatal suture in the axial plane was assessed by calculating the interdigitation index, a comparison of the sutural distance (μm) to linear sutural distance (μm).

Angelieri et al [3] developed a novel qualitative methodology for individual evaluation of midpalatal suture maturation. Two evaluators defined the maturational stages (A-E) via comparison of the morphological description of the palatal suture found in previous histologic studies [13–15] to the appearance of the suture in the axial plane generated from a standardized CBCT protocol of 140 subjects during initial records [3] To assess the reliability of defining the maturational stages (A-E) a validation study utilizing 30 random axial CBCT cross-sections of the midpalatal suture was performed by three evaluators and weighted kappa coefficients calculated [3].

Kwak et al [12] utilized an objective and quantitative method of fractal analysis, a methodology established previously for the evaluation of mammalian cranial sutures, [16] to be used for the first time in conjunction with CBCT imaging to evaluate the maturity of the midpalatal suture [12]. The cross-sectional study involved 131 subjects (69 men and 62 women) with a mean age of 24.1 ± 5.9 years. Each subject underwent CBCT imaging, followed by significant image processing to evaluate Cervical Vertebrae Maturation (CVM) stage, palatal stage of maturation (A-E, as defined by Angelieri et al. [3]) and isolation of a ROI for the calculation of the fractal dimension of the palatal suture. To assess the intra- and inter-reliability of defining the maturational stages (A-E), 30 random axial CBCT cross-sections of the midpalatal suture were staged by two other evaluators under controlled conditions and weighted kappa coefficients calculated, analogous to the study by Angelieri et al. [3] Statistical analysis included utilizing Scheffe’s ANOVA to compare the fractal dimension for each individual maturation stage (A-E) and subsequent Spearman’s coefficient calculation to ascertain the correlation between fractal dimension and maturation stage. The generation of a receiver operating characteristic (ROC) curve was used to develop an optimal fractal dimension cut-off value and sensitivity, specificity, false positive rate, false negative rate, positive predictability, and negative predictability calculated. For all statistical analysis, results were considered statistically significant at *P* < 0.05 [12].

Sumer et al. [9] utilized US to evaluate palatal sutural mineralization in three patients at five different time points; once after the 14 day surgically-assisted RME (SARME) expansion protocol, 2 months post-expansion, 4 months post-expansion, at time of removal of the tooth-borne expander (6 months post- expansion) and 2 months after appliance removal. The authors report that the ultrasound probe was used intra-orally on the skin that overlies the palatal suture, obtaining axial scans with the probe directed perpendicular to the length of the suture [9]. The authors assigned semi-quantitative bone fill scores (0–3). A bone fill score = 0 was characterized by open suture with clean gap margins and 0% echogenic material. A bone fill score = 1 was characterized by partial ultrasound transmission, localization of gap margins, and reduced echogenic material of ≤50%. A bone fill score = 2 was characterized by partial ultrasound transmission, marginally visible gap margins, and increased echogenic material of >50%. A bone fill score = 3 was characterized by no ultrasound transmission, 100% echogenic readings, and unidentifiable gap margins. The bone filling trends were qualitatively supported by comparison to conventional occlusal radiography [9].

### Synthesis of results

Due to high methodological heterogeneity among the included studies a meta-analysis was not supported.

### Risk of bias across studies

Each proposed technology or methodology to assess the maturation of the palatal suture lacked validation with a reference standard, namely histological evaluation. There was a lack of homogeneity in the quality of evidence amongst all five studies, ranging from an in-vitro study on human autopsy material [10] to human subject prospective studies [9, 11]. Sample sizes across all studies varied greatly, from 3 subjects [9] to a high of 140 subjects in a human subject cross-sectional study [3].

### Additional analysis

Not applicable due to lack of meta-analysis.

## Discussion

### Summary of evidence

#### Modality #1 – Multi-slice low-dose CT and quantitative bone density measurements (HU).

A technique to assess palatal suture maturation includes the use of multi-slice low-dose CT to capture axial slices of the maxilla and quantitatively measure the bone density at a particular ROI in HU [11]. It is known that CT is an excellent modality to evaluate the localized architecture of cancellous and cortical bone of the jaws; [17] however, less is known regarding the quantitative measurement of bone density, the HU scale. Hus were first utilized in dentistry to evaluate the pre-surgical bone density of implant sites [17–19]. The HU scale is a linear transformation of tissue attenuation coefficients where air is defined as −1000 HU, distilled water at standardized conditions equal to 0 HU and very dense bone defined as ≥1000 HU [17]. Consequently the authors considered and utilized the calculated Hus as an applicable unit of measurement to quantitatively assess mineralization at the palatal suture [11].

Franchi et al. [11] utilized the Houndsfield quantitative scale to evaluate the radiodensity of four previously mentioned ROI in the maxilla, 2 sutural and 2 bony areas. Pre-expansion (T0) statistical analysis noted a significant difference between the anterior and postural sutural regions (563.3 ± 183.29 HU and 741.7 ± 167.1 HU) and anterior and posterior bony areas (1057.5± 129.4 HU and 1102.8 ± 160.9 HU) (*P* < 0.05) (Table 2). Further statistical analysis yielded a significant difference between the anterior sutural and posterior sutural landmarks at T0 (*P* < 0.05), but no significant differences of these sutural areas at T1 or T2 (*P* > 0.05, Mann-Whitney). A significant difference between the radiodensity of the anterior and postural sutural ROIs between T0 and immediately post-expansion (T1), but no difference between their radiodensities when comparing pre-treatment (T0) and the post-expansion retention phase (T2) readings (*P* < 0.05) (Table 2).

Throughout the course of the study, trends in bone density measurements at the suture and its comparison to lateral bony sites followed conventional expectations of successful RME. Pre-expansion the measured HU at the anterior sutural region was significantly smaller than that of the posterior sutural site, and the applied expansion protocol introduced differential sutural opening with greatest opening at the anterior sutural region consistent with the pre-expansion HU scores. Additionally, the results measured at T2 at the end of the 6 month RME retention protocol, were congruent with previous histologic findings, namely post-expansion evidence of reorganization and sutural interdigitation [20].

An inherent advantage of using a low-dose CT protocol, where the voltage was decreased to 80 kV (KV), is subjecting the patient to a lower absorbed dose required for children undergoing radiologic evaluation [21]. Additionally, when the kilovoltage is reduced, image contrast of anatomical structures increases while still acceptable for assessing bone quality via this protocol [21]. Future areas of interest relating to the findings and protocol of this study would include further studies to define an anterior sutural HU: postural sutural HU ratio that best predicts the success of RME treatment. Conversely, further studies could elucidate specific ratios comparing sutural radiodensity to maxillary bony radiodensity that may predict an improved expansion prognosis.

It has to be noted that the reliability of using HU between subjects and within the same subjects the same day has not been demonstrated. Therefore some variation could be due to such factors. Also, not all studies specified patient orientation when taking the images thus effect of patient positioning on the image and HU or grey values is another aspect that should be tested.

#### Modality #2 – Micro-CT quantification of 3D palatal suture in the frontal and axial planes.

Korbmacher et al. [10] proposed assessing palatal suture maturation via micro-CT scanning and calculation of a number of developed indices, namely the obliteration index (%) and mean obliteration index (%) in the frontal plane, as well as, suture length [μm], linear sutural distance [μm] and interdigitation index in the axial plane.

Korbmacher et al. [10] evaluated 28 human palate specimens in the frontal and axial planes. In the frontal plane there was no demonstrated age dependent difference in the mean obliteration index between specimens (*P* = 0.244). The specimens were classified into one of three age groups (<25 years of age (yo), ≥25 to <30 yo and ≥30 yo) and results demonstrate that the frontal plane obliteration index varied across age groups between a minimum index of 0% to a maximum interdigitation of 7.3% (44 yo patient) (Table 2). Although the ≥25 to <30 yo age group consistently had a higher obliteration index in the frontal plane compared to other age groups, the results were not significant. Across all age groups, each subject had at least one frontal sutural cross-section that was devoid of interdigitation (mean obliteration index of 0%), with the oldest patient exhibiting a frontal plane mean obliteration index of 0% being a 71yo female. Investigation into the degree of interdigitation in the axial plane demonstrated no significant age-dependent differences in the calculated interdigitation index (*P* = 0.633). The authors did report a large standard deviation in the interdigitation index in the axial plane in the youngest and oldest age groups, and considerably less variation in the calculated index in the middle (<25 yo group and >30 yo) group [10] (Table 2).

Results indicated a generally low obliteration index amongst all subjects as well as an age-independent degree of interdigitation in the axial plane; however, across all measured indices there was significant intra-sutural and inter-subject variation [10]. This was the first time micro-CT was used on human samples and although this methodology was not implemented as part of an active expansion study, its principles can still be important to evaluate the pre-expansion maturity of the palatal suture. Additionally, it could be applied during mid-expansion protocol to evaluate the efficacy of treatment via calculation of the above noted indices and evaluation of the sutural architecture.

A limiting feature of the Korbmacher et al. [10] modality is the fact cadaver specimens were used, making direct translation of this study’s findings poorly applicable to clinical practice [15]. Considering the limitations of the gantry size of the micro-CT unit, and maximum scanning time used (200 min), micro-CT is best used on ex-vivo samples, and very small in-vivo samples to avoid an excessive absorbed dose emitted to patients [22]. Consequently, the use of micro-CT for in-vivo radiologic evaluation of the palate is impractical at this time. Therefore continued improvements to micro-CT technology including decreasing the emitted radiation while maintaining superior resolution, is necessary prior to implementation of such a technique on active RME patients.

An area of interest is the development of a CT-based strain assessment of peri-sutural and maxillary tissues; the development of which the authors believe will help facilitate predicting the success of RME treatment [10].

#### Modality #3 - US and assignment of semi-quantitative bone fill scores (0–3).

Sumer et al. [9] utilized US to evaluate sutural mineralization at five time points during the SARME and retention protocol for three patients, scoring each patient’s palatal suture calcification via assignment of semi-quantitative bone fill scores (0–3).

US findings in the Sumer et al. [9] study demonstrated that immediately post-expansion all subjects had a bone fill score = 0. (Table 2) Two of the three subjects at 2 and 4 months post-expansion were identified as having a bone score = 1, while the remaining subject was determined to have a bone fill score = 2 for these same time periods. Following the removal of the tooth-borne appliance at 6 months and 2 months subsequent to that during continued fixed appliance therapy, the bone scores for two of the subjects demonstrated increased mineralization and identification of echogenic material, having bone fill scores =2. The remaining patient received a bone fill score = 3 due to incomplete transmission of the waves and 100% echogenicity measured at these respective time points [9]. (Table 2) It should be noted that no statistics were reported by the authors.

The results of this study follow those of a similar animal study, [23] such that there was a statistically significant increase in bone fill scores that were directly related to the length of time the patient has been in retention post expansion. A major advantage to US is its low cost and non-invasiveness, [9, 23, 24] as well as improved usability compared to other methodologies, with the ability to perform real-time chair side evaluations with smaller hand held units. Additionally, US is a reliable method to image early bone formation as demonstrated by previous studies involving distraction osteogenesis [9, 23, 24]. A study comparing US to normal panoramic radiography, demonstrated that the efficacy of US to measure an osteotomy gap during distraction osteogenesis is equal to that of conventional radiography [9, 25]. US also demonstrated increased reliability compared to panoramic radiography to evaluate the maturation of early bone formation [9, 25] in the distraction gap. A disadvantage to US is its inability to penetrate cortical bone [9] However, following SARME or successful RME the osteotomy gap and its margins are easily visualized [9]. An area of significant future interest is to ascertain whether this technology can penetrate an immature midpalatal suture prior to the start of RME treatment, and allow the clinician to perform a chair side subjective evaluation of the bone maturity and interdigitation along the whole length of the suture. Limitations to this study included a very small sample size of three patients and lack of a gold standard (histology) or CT to validate the findings. Consequently, an area of future research is the use of this technology and bone fills scores in a similar larger sample size study in conjunction with a gold standard methodology to support the findings [9].

#### Modality #4 - CBCT and proposed maturation stages.

Angelieri et al. [3] utilized a standardized methodology to capture axial CBCT cross-sections of the palatal suture to provide individual staging of midpalatal suture maturation from the authors’ proposed maturation stages (A-E).

As it relates to Angelieri et al. [3] a validation study performed reported a weighted Kappa statistic for intra- and interexaminer reliability to be κ =0.75 (95% Confidence Interval (CI), 0.64–0.99) and be κ =0.79 (95% CI, 0.60–0.97) (no *P*-value reported), respectively. Due to a lack of an histologic or micro-CT gold standard, the authors also reported examiner reliability compared to the “ground truth”, a descriptor used to represent consensus among examiners with the principal investigator’ radiographic evaluations or other interpretations. Examiner reliability with ground truth ranged from κ = 0.82 (95% CI, 0.64–0.99) to κ =0.93 (95% CI, 0.86–1.00) (no *P*-value reported) [3].

Results of the validation study demonstrated “almost perfect” inter-examiner reliability with the “ground truth”, however, the authors did not report appropriate *P*-values with their statistics. As was mentioned before, there was no reference standard utilized during the validation study, but rather utilized what the authors termed the “ground truth”, [3] the professional opinion of the principal investigator when utilizing their own proposed maturation stages to classify each patient’s sutural maturation. Due to the lack of a gold standard, nor listed *P*-values, the results of the validation should be interpreted with caution. An additional limitation of this methodology is the proposed novel palatal suture maturation classification system itself. The authors developed the stages (A-E) based on comparison of CBCT axial cross-sections of the palatal suture to the perceived likeness of this radiographic morphology to the histological morphology of the suture as determined by previous studies [13–15]. Theoretically direct comparison of the histological morphology to the CBCT morphology of the suture is incompatible due to the histological assessment being on the microscopic scale as compared to the macro or eye level scale of sutures depicted in the CBCT axial slices. Consequently, any inference or direct translation of the sutural histological appearance and subsequent development of CBCT based sutural maturation stages is not possible. Therefore, the findings and developed maturational stages should be used with caution, and should not drive clinical decision making. Rather, at best, this maturational staging may be used as part of an extended protocol to subjectively assess palatal suture maturity during the treatment planning process. Future studies to thoroughly validate the proposed maturation stages to an available gold standard are advised.

#### Modality #5 – CBCT and fractal analysis to quantitatively ascertain degree of sutural maturation per proposed maturation stages of Anglieri et al. [3]

Kwak et al. [12] utilized CBCT imaging in conjunction with quantitative fractal analysis to ascertain if this analysis can be correlated to the maturational stage of each subjects palatal suture. Conceptually fractal analysis is based on the observation that cranial sutures can be visualized as a fractal pattern, [16] the dimensions of which are directly related to localized stresses experienced [12]. Additionally, the closer the approximation of two articulating bones, the more complex sutural morphology [12] suggestive of a more mature suture. Conceptually sound, fractal analysis has demonstrated its applicability in various areas dental research [26].

Fractal dimension intra- and inter-reliability results from the Kwak et al. [12] study demonstrated agreement with calculated weighted kappa coefficients of 0.84 (95% CI 0.74–0.93) and 0.67 (95% CI 0.38–0.95) to 0.72 (95% CI 0.48–0.97), respectively (Table 2). The CVM index inter- and intra-examiner reliability demonstrated agreement with weighted kappa coefficients from 0.69 (95% CI 0.53–0.86) and 0.71 (95% CI 0.56–0.86), respectively. The authors reported that none of the patients investigated possessed a CVM 1-IV nor was any subject classified as having palatal suture maturational stage A. It was found that 13 of 21 subjects with CVM V were classified as having maturational stage B or C (61.9%; males 77.8%, females 50.0%). Additionally, 42 of 110 subjects with CVM VI were classified as having maturational stage B or C (38.2%; males 41.6%, females 34.0%). Post-hoc analysis demonstrated that maturational stages B, C, D and E were related to differences in mean fractal dimension (*P* < 0.05). A negative correlation existed between fractal dimension and maturation stage (−0.623, *P* < 0.001). Male and female correlation coefficients were determined to be −0.649 (*P* < 0.001) and −0.569 (*P* < 0.001) respectively. A ROC curve was generated and determined the boundary between dichotomous maturation stages A–C and D or E, allowing for fractal dimension to be used to identify midpalatal suture fusion. Predictive statistical analysis noted that fractal dimension is a statistically significant indicator capable of predicting dichotomous maturation stages ((A, B, & C) vs. (D or E) (area under ROC curve [AUC] = 0.794, *P* < 0.001) [12] (Table 2).

The study notes a significant correlation between fractal patterning and degree of maturation of the midpalatal suture, and consequently the authors feel that fractal analysis can provide an objective and quantitative methodology to assess palatal suture maturity [12].

Disadvantages of this methodology include requiring significant training and proficiency in classifying the maturation stage of palatal sutures as proposed by Angelieri et al. [3]. Another disadvantage is requiring the clinician to have significant familiarity with image processing and possessing necessary software. Consequently, the time, cost and resources to do so may be prohibitive to clinicians. Additionally, this modality relies on complex statistical analyses to determine the variable (optimal cut-off value) to predict the dichotomous maturation stage of the patient’s palatal suture. Kwak et al. [12] argue that if an individual’s fractal dimensions can be compared, it may provide a straightforward and clinically viable method to assess the maturation of the palatal suture and aid in clinical decision making as it relates to the modality of expansion at the diagnostic record visit [12]. Conversely, the authors do note a variety of methods to calculate fractal dimensions and the fact these varying techniques produce different fractal dimension values. Consequently, Kwak et al. [12] argue for a more agreed upon method for its calculation to be utilized clinically.

Performing and interpreting these analyses requires significant advanced knowledge of statistics. Ultimately it is the view of the authors that this methodology is impractical in terms of time, cost, resources and knowledge required to complete this methodology for each patient as part of their diagnostic work up in day-to-day clinical practice.

Furthermore, as was stated previously in the discussion, utilization of the crudely proposed maturational staging as defined by Angelieri et al. [3] should be used with caution and lacks validation to a reference standard as does this study as mentioned by Kwak et al. [12]. Further areas of interest include the development of a ratio comparing the fractal dimensions of a mature coronal suture to that of the midpalatal suture [12]. Additionally, improvement in the accuracy of the methodology may be gained by refinement and minimization of the number of actions needed to determine fractal dimensions [12].

### Limitations

As mentioned before significant methodological differences were identified (sample size, in vitro vs. in vivo, imaging technique used, lack of adequate reference standard). The results were non-homogenous consequently a meta-analysis could not be performed, nor direct comparison of the studies possible, limiting any major conclusions regarding these newer contemporary methodologies to assess midpalatal sutural maturation. Overall, these studies did not present solid evidence of their validity for the accurate determination of the maturation of the palatal suture. As a consequence of this weak body of evidence, it is of utmost importance that clinicians use a multitude of diagnostic criteria to properly direct clinical decision making as it pertains to the maturity of the mid palatal suture and appropriate modality of expansion, namely RME or SARME. It is worth noting that expansion does not solely involve the palatal suture but also the circummaxillary sutures, this would also be a limitation present.

## Conclusions

- Only a weak limited body of evidence exists to support the newest technologies and proposed methodologies that evaluate the extent of mid palatal suture maturation.
- All discussed novel methodologies lack validation with histological reference/gold standard. Consequently, it is still advised that clinicians use a multitude of diagnostic criteria to subjectively assess palatal suture maturation and drive clinical decision-making as it relates to the appropriate treatment of maxillary skeletal transverse deficiency in late adolescents and young adults (RME vs. SARME).
- Future considerations in the imaging and assessment of the midpalatal sutural maturation will likely include some form of invasive CT technology, and proposed methodologies should follow appropriate ALARA radiation safety protocols.
- Non-invasive imaging technologies such as ultrasound present a promising and biologically safer alternative to assess midpalatal sutural ossification.

## Additional file

Additional file 1: Articles excluded from final inclusion criteria and reasons. (DOCX 17 kb)

## Abbreviations

CBCT: Cone-beam computed tomography
CT: Computed tomography
2D: Two-dimensional
3D: Three-dimensional
RME: Rapid maxillary expansion
SARME: Surgically assisted rapid maxillary expansion
US: Ultrasonography
PRISMA: Preferred Reporting Items for Systematic Reviews and Meta-Analysis
ROI: Region of Interest
HU: Hounsfield Units
CVM: Cervical Vertebrae Maturation
ROC: Receiver Operating Characteristic
T0: Pre-expansion
T1: Post-expansion
T2: Retention phase
KV: Kilovolts
CI: Confidence interval

## References

[1] McNamara JA (2000). Maxillary transverse deficiency. Am J Orthod Dentofac Orthop.

[2] Bishara SE, Staley RN (1987). Maxillary expansion: clinical implications. Am J Orthod Dentofac Orthop.

[3] Angelieri F, Cevidanes LH, Franchi L, Gonçalves JR, Benavides E, McNamara JA (2013). Midpalatal suture maturation: classification method for individual assessment before rapid maxillary expansion. Am J Orthod Dentofac Orthop.

[4] Isiksal E, Hazar S, Akyalcin S (2006). Smile esthetics: perception and comparison of treated and untreated smiles. Am J Orthod Dentofac Orthop.

[5] Lin L, Ahn HW, Kim SJ, Moon SC, Kim SH, Nelson G (2015). Tooth-borne vs bone-borne rapid maxillary expanders in late adolescence. Angle Orthod.

[6] Baccetti T, Franchi L, Cameron CG, McNamara JA (2001). Treatment timing for rapid maxillary expansion. Angle Orthod.

[7] Melsen B (1975). Palatal growth studied on human autopsy material. A histologic microradiographic study. Am J Orthod.

[8] Persson M, Thilander B (1977). Palatal suture closure in man from 15 to 35 years of age. Am J Orthod.

[9] Sumer AP, Ozer M, Sumer M, Danaci M, Tokalak F, Telcioglu NT (2012). Ultrasonography in the evaluation of midpalatal suture in surgically assisted rapid maxillary expansion. J Craniofac Surg.

[10] Korbmacher H, Schilling A, Püschel K, Amling M, Kahl-Nieke B (2007). Age-dependent three-dimensional microcomputed tomography analysis of the human midpalatal suture. J Orofac Orthop.

[11] Franchi L, Baccetti T, Lione R, Fanucci E, Cozza P (2010). Modifications of midpalatal sutural density induced by rapid maxillary expansion: a low-dose computed-tomography evaluation. Am J Orthod Dentofac Orthop.

[12] Kwak KH, Kim SS, Kim YI, Kim YD (2016). Quantitative evaluation of midpalatal suture maturation via fractal analysis. Korean J Orthod.

[13] Persson M, Magnusson BC, Thilander B (1978). Sutural closure in rabbit and man: a morphological and histochemical study. J Anat.

[14] Cohen MM (1993). Sutural biology and the correlates of craniosynostosis. Am J Med Genet.

[15] Sun Z, Lee E, Herring SW (2004). Cranial sutures and bones: growth and fusion in relation to masticatory strain. Anat Rec A Discov Mol Cell Evol Biol.

[16] Yu JC, Wright RL, Williamson MA, Braselton JP, Abell ML (2003). A fractal analysis of human cranial sutures. Cleft Palate Craniofac J.

[17] Shapurian T, Damoulis PD, Reiser GM, Griffin TJ, Ran WM (2006). Quantitative evaluation of bone density using the Hounsfield index. Int J Oral Maxillofac Implants.

[18] Duckmanton NA, Austin BW, Lechner SK, Klineberg IJ (1994). Imaging for predictable maxillary implants. Int J Prosthodont.

[19] Norton MR, Gamble C (2001). Bone classification: an objective scale of bone density using the computerized tomography scan. Clin Oral Implants Res.

[20] Cleall JF, Bayne DI, Posen JHM, Subtelny JD (1965). Expansion of the midpalatal suture in the monkey. Angle Orthod.

[21] Ballanti F, Lione R, Fanucci E, Franchi L, Baccetti T, Cozza P (2009). Immediate and post-retention effects of rapid maxillary expansion investigated by computed tomography in growing patients. Angle Orthod.

[22] Perilli E, Parkinson IH, Reynolds KJ (2012). Micro-CT examination of human bone: from biopsies towards the entire organ. Ann Ist Super Sanita.

[23] Thurmüller P, Troulis M, O'Neill MJ, Kaban LB (2002). Use of ultrasound to assess healing of a mandibular distraction wound. J Oral Maxillofac Surg.

[24] Hughes CW, Williams RW, Bradley M, Irvine GH (2003). Ultrasound monitoring of distraction osteogenesis. Br J Oral Maxillofac Surg.

[25] Bruno C, Minniti S, Buttura-da-Prato E, Albanese M, Nocini PF, Pozzi-Mucelli R (2008). Gray-scale ultrasonography in the evaluation of bone callus in distraction osteogenesis of the mandible: initial findings. Eur Radiol.

[26] Sánchez I, Uzcátegui G (2011). Fractals in dentistry. J Dent.

