# Supplementary material for: Novel methodologies and technologies to assess mid-palatal suture maturation: a systematic review
Source: Head Face Med. 2017 Jun 14;13:13. doi: 10.1186/s13005-017-0144-2 (PMC5471738; doi:10.1186/s13005-017-0144-2)
Supplement: Additional file 1: — Articles excluded from final inclusion criteria and reasons. (DOCX 17 kb) [file 13005_2017_144_MOESM1_ESM.docx]

Appendix 2: Articles excluded from final inclusion criteria and reasons

| **Author(s)** | **Reason Excluded** |
| --- | --- |
| Fricke-Zech^1^ | Animal study (porcine model) using flat-panel volume CT (fpVCT) |
| Gao^2^ | Animal study (dog) using transmission electron microscope (TEM) |
| Hahn^3^ | Animal study (pig) using fpVCT and multislice-computed tomography (MSCT) |
| Beauthier^4^ | Methodology is not novel, used in forensics for age of death using maxilla |
| Cheung^5^ | Animal study (Rhesus monkeys) using conventional radiography and microcomputerized scanning |
| De Melo Mde^6^ | Study used conventional digital radiography |
| Gurgel jde^7^ | Study used conventional radiography that was later digitized |
| Kjaer^8^ | Study used conventional radiography |
| Knaup^9^ | Study used established histological analysis |
| Lee^10^ | Study used conventional radiography and histological analysis |
| Leonardi^11^ | Study evaluated spheno-occipital synchondroses displacement during RME not midpalatal suture using multislice multidetector CT |
| Leonardi^12^ | Study evaluated circumaxillary sutures during RME not midpalatal suture using multislice multidetector CT |
| Kjaer^13^ | Study used conventional radiography and histological analysis |
| Agrawal^14^ | Study used conventional radiography to evaluate patency of cranial suture following surgical treatment of cranial synostosis |
| Bradley^15^ | Animal study (mouse) that utilized conventional histological analysis |
| Captier^16^ | Study evaluated sphenofrontal suture with light microscopy |
| Lauridsen^17^ | Study used established histological analysis |
| Corega^18^ | Study assessed coronal cranial sutures using Micro-CT |
| Corega^19^ | Study assessed coronal cranial sutures using Micro-CT |
| Bjork^20^ | Article in German, no translation services available. |
|  |  |
| Sannomiya^21^ | Study used conventional radiography |
| Takenouchi^22^ | Animal study (rats) utilizing in vivo micro-computed tomography (mCT). |
| Acar^23^ | Study used previously described novel technique using Hounsfield units to assign bone density at the palatal suture. |

**References for Appendix**

1. Fricke-Zech S, Gruber RM, Dullin C, et al., Measurement of the midpalatal suture width. Angle Orthod 2012;82:145-50.

2. Gao QW, Chai JK, Song HF, Xu MH, Jing S, Liu CM. [An ultrastructure study on the palatomaxillary suture of dog expanded by NiTi-SMA]. Zhonghua Zheng Xing Wai Ke Za Zhi 2009;25:277-9.

3. Hahn W, Fricke-Zech S, Fialka-Fricke J, et al. Imaging of the midpalatal suture in a porcine model: flat-panel volume computed tomography compared with multislice computed tomography. Oral Surg Oral Med Oral Pathol Oral Radiol Endod 2009;108:443-9.

4. Beauthier JP, Lefevre P, Meunier M et al. Palatine sutures as age indicator: a controlled study in the elderly. J Forensic Sci 2010;55:153-8.

5. Cheung LK, Zhang Q. Radiologic characterization of new bone generated from distraction after maxillary bone transport. Oral Surg Oral Med Oral Pathol Oral Radiol Endod 2003;96:234-242.

6. de Melo Mde F, Melo SL, Zanet TG, Fenvo-Pereira M. Digital radiographic evaluation of the midpalatal suture in patients submitted to rapid maxillary expansion. Indian J Dent Res 2013;24:76-80.

7. Gurgel Jde A, Malmström MF, Pinzan-Vercelino CR. Ossification of the midpalatal suture after surgically assisted rapid maxillary expansion. Eur J Orthod 2012;34:39-43.

8. Kjaer I. Human prenatal palatal shelf elevation related to craniofacial skeletal maturation. Eur J Orthod 1992;14:26-30.

9. Knaup B, Yildizhan F, Wehrbein H. Age-related changes in the midpalatal suture. A histomorphometric study. J Orofac Orthop 2004;65:467-74.

10. Lee SK, Kim YS, Lim CY, Chi JG. Prenatal growth pattern of the human maxilla. Acta Anat (Basel) 1992;145:1-10.

11. Leonardi R, Cutrera A, Barbato E. Rapid maxillary expansion affects the spheno-occipital synchondrosis in youngsters. A study with low-dose computed tomography. Angle Orthod 2010;80:106-10.

12. Leonardi R, Sicurezza E, Cutrera A, Barbato E. Early post-treatment changes of circumaxillary sutures in young patients treated with rapid maxillary expansion. Angle Orthod 2011;81:36-41.

13. Kjaer I. Prenatal skeletal maturation of the human maxilla. J Craniofac Genet Dev Biol 1989;9:257-64.

14. Agrawal D, Steinbok P, Cochrane DD. Reformation of the sagittal suture following surgery for isolated sagittal craniosynostosis. J Neurosurg 2006;105: 115-7.

15. Bradley JP, Levine JP, Roth DA, McCarthy JG, Longaker MT. Studies in cranial suture biology: IV. Temporal sequence of posterior frontal cranial suture fusion in the mouse. Plast Reconstr Surg 1996;98:1039-45.

16. Captier G, Cristol R, Montoya P, Prudhomme M, Godlewski G. Prenatal organization and morphogenesis of the sphenofrontal suture in humans. Cells Tissues Organs 2003;175:98-104.

17. Lauridsen H, Fischer Hansen B, Reintoft I, Keeling JW, Kjaer I. Histological investigation of the palatine bone in prenatal trisomy 21. Cleft Palate-Craniofac J 2001;38:492-7.

18. Corega C, Vaida L, Băciuţ M, Serbănescu A, Palaghiţă-Banias L. Three-dimensional cranial suture morphology analysis. Rom J Morphol Embryol 2010; 51:123-7.

19. Corega C, Vaida L, Iliaş IT, Bertossi D, Dascălu IT. Cranial sutures and diploae morphology. Rom J Morphol Embryol 2013;54:1157-60.

20. Björk A, Skieller V. [Growth and development of the maxillary complex]. Inf Orthod Kieferorthop 1984;16:9-52.

21. Sannomiya EK, Macedo MM, Siqueira DF, Goldenberg FC, Bommarito S. Evaluation of optical density of the midpalatal suture 3 months after surgically assisted rapid maxillary expansion. Dentomaxillofac Radiol 2007;36:97-101.

22. Takenouchi H, Mayahara K, Arai Y, Karasawa Y, Shimizu N. Longitudinal quantitative evaluation of the mid-palatal suture after rapid expansion using in vivo micro-CT. Arch Oral Biol 2014;59:414-23.

23. Acar YB, Motro M, Erverdi AN. Hounsfield Units: a new indicator showing maxillary resistance in rapid maxillary expansion cases? Angle Orthod 2015;85:109-16.
